# Dynamic similarity promotes interpersonal coordination in joint action

**DOI:** 10.1098/rsif.2015.1093

**Published:** 2016-03

**Authors:** Piotr Słowiński, Chao Zhai, Francesco Alderisio, Robin Salesse, Mathieu Gueugnon, Ludovic Marin, Benoit G. Bardy, Mario di Bernardo, Krasimira Tsaneva-Atanasova

**Affiliations:** 1 Department of Mathematics, College of Engineering, Mathematics and Physical Sciences, University of Exeter, Exeter EX4 4QF, UK; 2 Department of Engineering Mathematics, University of Bristol, Merchant Venturers' Building, Bristol BS8 1UB, UK; 3 EuroMov, Montpellier University, 700 Avenue du Pic Saint-Loup, 34090 Montpellier, France; 4 Institut Universitaire de France, Paris, France; 5 Department of Electrical Engineering and Information Technology, University of Naples Federico II, 80125 Naples, Italy

**Keywords:** movement dynamics, statistical analysis, mathematical modelling

## Abstract

Human movement has been studied for decades, and dynamic laws of motion that are common to all humans have been derived. Yet, every individual moves differently from everyone else (faster/slower, harder/smoother, etc.). We propose here an index of such variability, namely an individual motor signature (IMS) able to capture the subtle differences in the way each of us moves. We show that the IMS of a person is time-invariant and that it significantly differs from those of other individuals. This allows us to quantify the dynamic similarity, a measure of rapport between dynamics of different individuals' movements, and demonstrate that it facilitates coordination during interaction. We use our measure to confirm a key prediction of the theory of similarity that coordination between two individuals performing a joint-action task is higher if their motions share similar dynamic features. Furthermore, we use a virtual avatar driven by an interactive cognitive architecture based on feedback control theory to explore the effects of different kinematic features of the avatar motion on coordination with human players.

## 1. Introduction

Humans often need to perform joint tasks and coordinate their movement [1]. Their motion can be studied and classified by means of some common generalized movement laws [2–4] that define a ‘human-like’ way of movement [5,6]. However, every individual moves following a specific personal style characterized by unique kinematic features. An open problem is to find methods that capture such features and identify different individuals from the way they move. This is key to show that individuals moving in similar ways exhibit higher levels of synchronization when performing joint tasks.

Specifically, studies of interpersonal interaction show that people prefer to interact with others who are similar to themselves [7,8]. Moreover, it has been shown that social movement coordination between interacting people could be used to assess and enhance their mutual rapport [9–12]. These observations have led to the development of a theory of similarity which predicts that the level of synchronization in joint actions is enhanced if the participants are similar in terms of morphology and movement dynamics and are willing to match their behaviours [13–15]. Despite previous attempts in the literature [16], the theory of similarity has not been tested in controlled experiments.

In this paper, we demonstrate existence of a time-invariant individual motor signature (IMS), and show how it can be used to study sociomotor coordination. The notion of IMS has its roots in the concept of frequency detuning (eigenfrequency difference) between two interacting humans and the phenomenon of the so-called maintenance tendency [17–19]. It has been further extended to intrinsic dynamics that is observed in the form of individual preferred coordination modes (behavioural repertoire) exhibited during intrapersonal coordination tasks [20,21]. We focus here on identifying a more general measure able to capture some key kinematic features of the motion of each individual and discriminate among different people. Using our measure, we are able to introduce a metric to assess the *dynamic similarity* between the movement of different humans and show that it helps to predict the level of coordination in an interactive joint-action task.

We demonstrate the theory by using a virtual avatar playing the ‘mirror game’, an activity where two players are asked to imitate each other's movements, and which has recently been established as a paradigm for studying interpersonal movement dynamics (see [22,23] and references therein). Our evidence shows that similarity between the preferred motion of each player enhances the synchronization level measured during the interaction.

More generally, our results introduce dynamic similarity as an important, complementary to qualitative measures of affiliation, factor affecting joint actions and enhancing coordination between socially interacting people.

## 2. Methods

The results presented in this paper are based on the analysis of data collected in three different experimental scenarios (each with different group of participants), performed in the course of the research project AlterEgo supported by the European Union [24]. In scenario 1, we collected only solo movements of the participants; in scenario 2, we collected data from humans playing the mirror game in a solo condition and in dyads where they have to track each other's movements; in scenario 3, we collected data from human participants playing solo and interacting with a virtual player (VP). Data collected in scenario 1 were used to establish existence of the IMS. Data collected in scenarios 2 and 3 are used to demonstrate that coordination during an interactive task depends on the dynamic similarity between participants. In particular, we use solo data collected in scenarios 2 and 3 to measure dynamic similarity between interacting participants.

### 2.1. Experimental set-up and data collection

In scenario 1, participants were asked to perform three solo sessions, each one separated by at least one week. Each participant was asked to sit comfortably on a chair and create interesting motion by moving her/his preferred hand above a leap motion^®^ sensor [25] connected to a laptop. The movement of a participant was visualized on the screen of the laptop as a dot. Participants were given the following instruction: ‘Play the game on your own, create interesting motions and enjoy playing’. Owing to the nature of the experimental set-up, the position was recorded in arbitrary units. At each session, a participant was required to perform three solo rounds, each one lasting 60 s. In total, we recorded nine position timeseries for each of the 15 participants.

In scenario 2, participants sat comfortably opposite each other. Two horizontal strings (length 1800 mm) were mounted at eye level, centrally between the participants; on each string, a ball with a small handle was mounted. Participants were instructed to move these balls left and right along the strings during the experiment. The movements of each participant were captured using reflecting markers placed on the ball with infrared MX13 cameras (Vicon-Nexus, Oxford Metrics Ltd) at a sampling rate of 100 Hz. Data were collected from eight dyads (16 participants in total). All participants were right handed. Participants were given the following instructions:

— *Solo condition*. Participants were given the same instruction as in scenario 1. Participants had no view of their partner.
— *Leader–follower condition*. ‘This is a collaborative round whose purpose is to enjoy creating synchronized motion. Participant 1, lead the movement. Participant 2 try to follow your partner's movement’. Two versions of this condition were played to allow both participants to lead and to follow.
— *Joint improvisation condition*. ‘In this collaborative round, there is no leader and no follower. Let these two roles emerge naturally, imitate each other and create synchronized and interesting motions. Enjoy playing together’.

In scenario 3, human players were asked to play with the VP described in [26,27]. Participants were standing in front of an LCD display showing the VP. A horizontal string (length 1800 mm) was mounted in front of the participant. As in scenario 2, a ball with a small handle was mounted on the string. Participants were instructed to move the ball left and right along the string. On the screen facing the human player, a ball, which is controlled by the VP, is also shown to move along a string. The movement of each participant was recorded with a single wide-angle camera. The sampling rate was not uniform and averaged around 40 Hz.

The VP was driven by an interactive cognitive architecture (ICA) which used a pre-recorded reference motion trajectory (Ref) and an adaptive feedback control algorithm to generate the VP's movement, while being influenced by the follower's performance (see [26,27] for further details). It is important to note that the ICA does not simply replay pre-recorded timeseries as in [28], but uses them as the preference signal in order to generate the output trajectory for the VP. This allows for a real-time movement behaviour matching between human and VPs, which is a fundamental part of the interaction in the mirror game. For instance, if the follower stops tracking the movement of the leader, then it is appropriate for the leader, as done by the ICA driving the VP, to adjust its movement and guide the follower in order to encourage the interaction. In scenario 3, the ICA driving the VP was fed with pre-recorded position timeseries based on solo trials of the human participant playing with it. More specifically, to control similarity between the solo motion and reference trajectory the pre-recorded solo trajectory of the player was superimposed with a 2.5 Hz sinusoidal signal with time-varying amplitude defined as one-third of the corresponding normalized velocity of the solo trajectory. Further analysis of the VP's performance can be found in the electronic supplementary material, §6. Data were collected from 51 individuals playing the mirror game with the VP. The dataset of each participant contained participant's and VP's positions for each of the following rounds: 4 solo (1 min) rounds (without VP) and 12 rounds (30 s) where the human participant played as a follower.

### 2.2. Data processing

The collected data were pre-processed in Matlab^®^. When necessary, we used interpolation with shape-preserving piecewise cubic interpolation and filtering with a zero-phase forward and reverse digital second-order lowpass (10 Hz cut-off) Butterworth filter. The position timeseries were then used to numerically estimate their corresponding velocity timeseries. To differentiate position timeseries, we used a fourth-order finite difference scheme. We cut out the first and last 2 s of the signal. Furthermore, we limited velocities to 3.5 (arb. units s^−1^) in experimental scenario 1 and to 2.7 (m s^−1^) in experimental scenarios 2 and 3 (higher velocities were considered as results of noise in the collected data). To estimate the probability density function (PDF) of the player's velocity, we use a normalized histogram of the velocity timeseries with 101 equally distant bins between −2.7 and 2.7 (m s^−1^) (or −3.5 and 3.5 (arb. units s^−1^) in scenario 1). Further details about data processing can be found in the electronic supplementary material, §1.

In order to quantify the similarity between PDFs of a player's velocity, we use the Earth mover's distance (EMD) that is an established tool in pattern recognition applications [29,30]. Intuitively, the EMD measures how much work is required to transform a ‘pile of earth’ into another; each ‘pile of earth’ representing a histogram. In the case of univariate probability distributions, the EMD is given by the area of the difference between their cumulative distribution functions. More details can be found in appendix A.

Distances between PDFs are then analysed by means of multidimensional scaling (MDS). MDS is a well-established tool in data visualization and data mining [31]. It allows to reduce dimensionality of the data and visualize relations between the objects under investigation while preserving as much information as possible. Because the EMD is a metric in the space of the PDFs of velocity timeseries, we use classical MDS in the form implemented in Matlab. Description of the MDS algorithm and discussion of relation between MDS and principal component analysis can be found in the electronic supplementary material, §3.

In order to quantify temporal correspondence (level of coordination) between players, we introduce the *relative position error* (RPE). The RPE is a measure of temporal correspondence between complex, non-periodic, coordinated movements which is based on the natural notion of a follower lagging behind the leader when tracking her/his motion. In particular, RPE is a measure of position mismatch between a leader and a follower capturing how well the follower tracks the leader's movement. See appendix C for further details. Discussion of the advantages of using the RPE over the relative phase based on the Hilbert transform can be found in the electronic supplementary material, §9 and §10.

## 3. Results

We begin by showing the existence of an IMS for each player, defined as a time-invariant-tractable characteristic of her/his movement. We develop a framework allowing us to demonstrate that characteristics of the solo movement in the mirror game are time-persistent and differ significantly between participants. In the proposed framework, we employ *velocity profiles* (PDFs of the velocity timeseries) to reveal that the rapport (similarity) between IMS enhances synchronization of movement between participants in joint action. Finally, we demonstrate that a VP driven by a novel ICA [26,27] can be used to study interpersonal interaction in a ‘mirror game’ between human and VPs.

### 3.1. Existence of an individual motor signature

Here, we study solo mirror game recordings collected in experimental scenario 1 in order to investigate the existence of an *IMS*. In particular, we demonstrate that (i) the movement characteristics of each individual persist in time and (ii) that they differ significantly between individuals.

To this end, we analyse *velocity profiles* that characterize motion in the mirror game on the timescale of a complete experimental trial. We use the EMD to assess distances between velocity profiles of different individuals. We then represent them as points in the *similarity space*, that is an abstract geometrical space constructed by means of MDS [31] that provides a visual representation of the pattern of proximities (i.e. similarities or distances) among a set of objects. As a result, we obtain clusters of points corresponding to solo trials of individual participants. In order to measure separation between clusters of points in the similarity space, we measure overlap *ω* between ellipses that encircle them, with *ω* = 0 meaning that the ellipses do not overlap at all and *ω* = 1 meaning complete overlap (see the electronic supplementary material, §5, for further details about ellipses and overlap).

**Figure 1.**
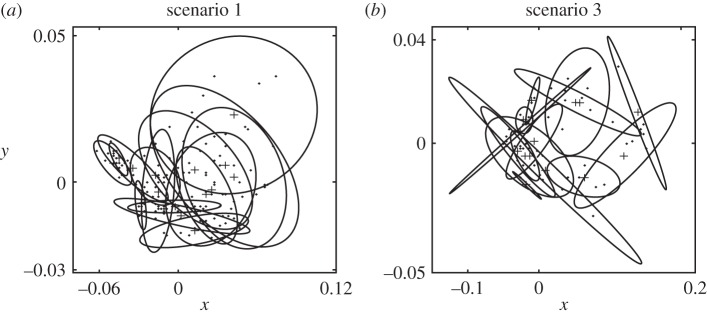
Individual motor signature in the *similarity space* computed with MDS from distances between velocity profiles. (*a*) For 15 different participants from solo mirror game recordings in scenario 1, on three different days with at least one week break between recording rounds. (*b*) For 56 solo trials of 14 participants from solo mirror game recordings in scenario 3 (for the sake of clarity data for only 14 of 51 participants is shown). Each ellipse corresponds to a different participant. Small dots correspond to individual solo recordings. Each cross at the centre of an ellipse corresponds to the average of the small dots' positions. Each ellipse indicates 0.7 mass of bivariate normal distribution fitted to the small dots (see the electronic supplementary material, §5 for further details).

Figure 1 depicts velocity profiles from solo trials presented as elements of the *similarity space*. Figure 1*a* shows data for 15 different participants from experimental scenario 1 and figure 1*b* shows representative data of 14 of 51 participant from experimental scenario 3. We note that, in experimental scenario 3, players had a larger range of movement, and all the solo trials of individual players were recorded on a single day. Each dot in figure 1 corresponds to a velocity profile from a single trial. The dots corresponding to different individuals are encircled by ellipses. Importantly, figure 1*a* demonstrates clustering of the dots for different participants collected on three different days. Such clustering indicates time-invariance of the IMS. The variability between radii of the ellipses associated with different individuals signifies that the IMS of some individuals is more variable than the others.

Furthermore, both datasets presented in figure 1 demonstrate a good separation of the ellipses corresponding to individual participants, with a median overlap *ω* between 15 ellipses in figure 1*a* equal to 0.02 and between ellipses of all 51 participants from experimental scenario 3 equal to 0.05. Interestingly, in the data from experimental scenario 1, there are 45 of 105 pairs of ellipses that do not overlap at all, meaning that in almost half of the cases two participants can be explicitly distinguished just by observing their solo motion. The same holds true for 418 of 1275 pairs of ellipses from experimental scenario 3. Comparison of separation between individuals achieved by means of the velocity profiles and using individual characteristic of motion suggested in [22,23,28] can be found in the electronic supplementary material, §5.

The physical interpretation of the two principal dimensions of the similarity space constitutes a further insight gained from our analysis. In particular, our analysis reveals that the coordinate *x* of the movement representation in the similarity space, which corresponds to the first principal dimension given by the MDS, is correlated with the absolute average velocity of the motion. In addition, the *y*-coordinate of the representation of each timeseries in the similarity space, which corresponds to the second principal dimension from the MDS, is correlated with the kurtosis of a velocity segment [22,23], a part of the velocity timeseries between two consecutive times of zero velocity. That is, it informs us on the ratio of high and low velocities in the motion. For further details about interpretation of the dimensions in the similarity space, see appendix B.

In summary, IMS identifies each different participant and can be used effectively to measure dynamic similarity between them. More importantly, it provides a comprehensive and holistic description of the kinematic characteristics and variability of human movement.

### 3.2. Behavioural plasticity during social interaction

**Figure 2.**
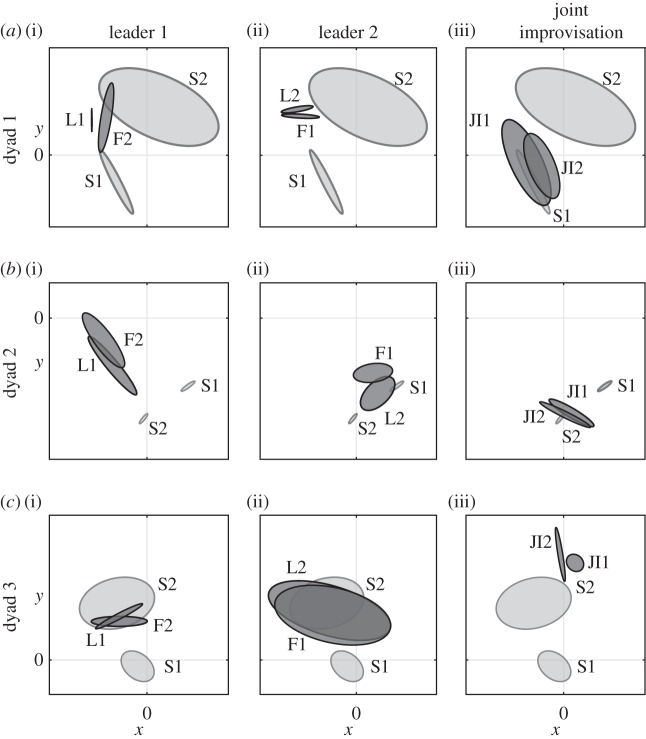
Interaction between two players in different experimental conditions visualized in the similarity space. Ellipses encircle points corresponding to velocity profiles in solo (S1 and S2; light grey), leader (L1 and L2; dark grey), follower (F1 and F2; dark grey) and joint improvisation (JI1 and JI2; dark grey) rounds. Each row depicts data for a different dyad. In column 1, player 1 was a leader, in column 2, player 2 was a leader and in column 3, participants played in joint improvisation condition. *x*-Axis has the same range in all panels, *y*-axis is rescaled for clarity of presentation.

Using the concepts of *IMS* and *similarity space*, we are able to demonstrate behavioural plasticity during social interaction. Specifically by behavioural plasticity, we mean that in order to cooperate people are willing, to a different degree, to disregard their individual preferences. Indeed, by comparing positions in the similarity space of the velocity profiles from solo and cooperative trials (leader–follower and joint-improvisation), we find that some people are more inclined to adjust their kinematic characteristics when interacting with others in the mirror game. Figure 2 shows three representative examples of the behavioural plasticity detected during the experimental scenario 2. In figure 2*a*(i) and *a*(ii), we depict consistent behaviour of the leader and follower independently of which player is the designated leader. In figure 2*b*(i) and *b*(ii), we note that dynamics of movement differs significantly depending on who is leading. In figure 2*c*(i) and *c*(ii), we illustrate a player (S2) that dominates the interaction in terms of movement characteristics. Furthermore, figure 2*a*(iii)–*c*(iii) shows that motion dynamics in the joint improvisation condition is clearly different compared with the leader–follower condition in agreement with recently published results [22]. Visualization of the interactions for all the eight dyads from the experimental scenario 2 can be found in the electronic supplementary material, figure S4.

Taken together, the above observations suggest that the behavioural plasticity might be regulable rather than fixed and could be modulated in order to enhance social competence. Figure 2 shows a new technique that could be useful in studies of mutual rapport, affiliation and leadership emergence. However, rigorous analysis of the complex relation between IMS and movements during cooperative conditions is beyond the scope of this paper.

### 3.3. Dynamic similarity enhances coordination in joint action

**Figure 3.**
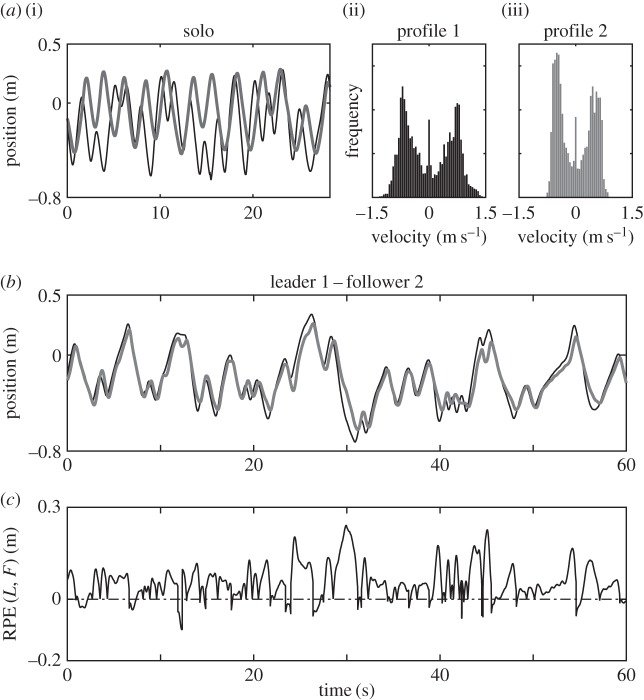
First, we compute dynamic similarity between two players. In panel (*a*(i)), we show the solo movements of two participants who later played together in the leader–follower condition. Panels (*a*(ii)) and (*a*(iii)) depict velocity profiles that represent individual motor signatures of the two players (S*_a_*_(ii)_, and S*_a_*_(iii)_) corresponding to the positions timeseries presented in panel (*a*(i)). The EMD(S_a(ii)_, S_a(iii)_) = 0.0303 between the histograms in panels (*a*(ii)) and (*a*(iii)) quantifies dynamic similarity between the two players. Then, we measure temporal correspondence between their movements when they play together in the leader–follower condition. Panel (*b*) illustrates position traces of the participants from panel (*a*) when they play together as a leader (black) and follower (grey). Panel (*c*) shows the RPE between leader and follower trajectories presented in panel (*b*). The mean value and the standard deviation of the RPE are respectively *μ*RPE(*L_a_*_(ii)_, *F_a_*_(iii)_) = 0.05 and *σ*RPE(*L_a_*_(ii)_, *F_a_*_(iii)_) = 0.05.

Having defined suitable measures, we next analyse the effects of the dynamic similarity on the temporal correspondence in two different experimental scenarios: the former where two humans play the mirror game (scenario 2) and the latter where a human is asked to play the game with a VP (scenario 3). In particular, we measure the temporal correspondence between players in the leader–follower condition of the mirror game and study systematically if and how it is related to the difference between their IMS. In order to demonstrate that dynamic similarity facilitates coordination between players in the mirror game, we seek to find a correlation between dynamic similarity, as quantified by means of the distance between velocity profiles in the similarity space, and temporal correspondence measured by the RPE. For further details about the definition and interpretation of the RPE, see appendix C. Figure 3*a*–*c* illustrates the steps we take in our analysis detailed in the figure legend.

**Figure 4.**
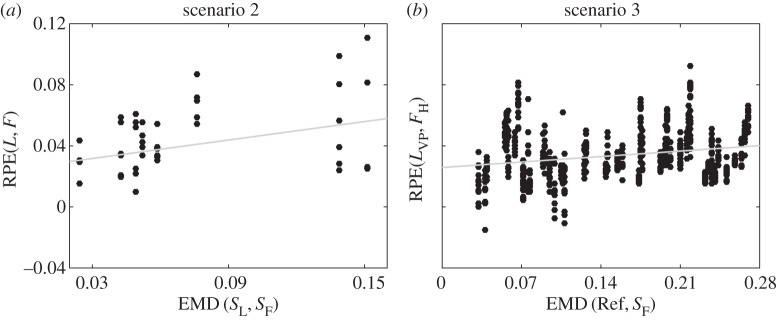
Panel (*a*) shows correlation between EMD(*S*_L_, *S*_F_) and RPE(*L*, *F*) computed for all individual leader–follower trials in eight dyads from scenario 2. Panels (*b*) depicts the dependence of RPE(*L*_VP_, *F*_H_) between the VP leading the human participant on EMD(Ref, *S*_F_) between the reference trajectory and the participant's solo movement (scenario 3). Each black dot corresponds to a single leader–follower trial. Grey lines are presented only for illustrative purposes. Spearman's *ρ* coefficients are equal to: *ρ_a_* = 0.3907 (*p_ρa_* = 7×10^−3^), *ρ_b_* = 0.2224 (*p_ρb_*<1 × 10^−5^); Pearson's *R*^2^ coefficients are equal to: 
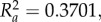
 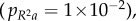 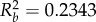 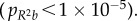

The correlation between temporal correspondence and dynamic similarity observed in the data from human–human interaction collected in the experimental scenario 2 is shown in figure 4*a*. For each dyad, we calculate nine values for the distance between the players signatures *S*_L_ and *S*_F_ (all the combinations between three solo trials for each player) and six values for the mean RPE between the leader (L) and the follower (F) (three trials with player 1 as a leader and three trials with player 2 as a leader); Spearman's rank correlation [32,33] between EMD distance and RPE was computed to be *ρ* = 0.3907 (*p_*ρ*_* = 7 × 10^−3^)). We remove a single outlier with RPE > 0.2 (the correlation including the outlier was stronger). We use Spearman's rank correlation, because our data are not normally distributed.

We further control the identified correlation for two confounding factors. First, we expect that a faster leader is more difficult to follow than a slower one, hence we control the identified correlation for the mean absolute velocity of the leader *μ*|*V*_L_|. Second, we expect that it is easier for a follower who prefers fast motions to track the movement of a leader who prefers to move slowly than it is for a follower who prefers slow motions to track a leader who prefers to move fast. Therefore, we control the identified correlation for the difference between mean absolute velocities of the solo movements of the leader and the follower *μ*|*V*_SL_| − *µ*|*V*_SF_|, which is proportional to the difference between their *x*-coordinates (see appendix B). Partial and adjusted correlation coefficients are computed in Matlab using functions partialcorr and partialcorri. As expected, we find the mean RPE between leader (L) and follower (F) to be strongly correlated with both *μ*|*V*_L_| and *μ*|*V*_SL_| − *μ*|*V*_SF_|. Nonetheless, the relation between mean RPE(*L, F*) and EMD(*S*_L_, *S*_F_) controlled for the confounding factors remains statistically significant; partial Spearman's rank correlation controlled for *μ*|*V*_L_| was computed to be *ρ* = 0.3466 (*p_*ρ*_* = 2 × 10^−2^); partial Spearman's rank correlation controlled for *μ*|*V*_SL_| − *μ*|*V*_SF_| was computed to be *ρ* = 0.5025 (*p_*ρ*_* = 3 × 10^−4^); Spearman's rank correlation adjusted for both, *μ*|*V*_L_| and *μ*|*V*_SL_| − *μ*|*V*_SF_|, was computed to be *ρ* = 0.4697 (*p_*ρ*_* = 1 × 10^−3^).

We confirm our observations by also analysing data from experimental scenario 3, where a VP leads a human follower. In particular, we compute correlations between the similarity of the two players' motions (evaluated in terms of the average distance between the velocity profile of the VP reference trajectory (Ref) and the four velocity profiles of the solo motion of the human player) and their temporal correspondence. Figure 4*b* demonstrates that temporal correspondence depends on the dynamic similarity between the reference trajectory of the VP and participant's solo movement. In particular, we find that the mean of the RPE between leader (*L*_VP_) and follower (*F*_H_) increases with the distance between their signatures. This finding affirms that dynamic similarity between reference trajectory and player's solo movements facilitates coordination between the VP leader and human (H) follower; the Spearman's rank correlation was computed to be *ρ* = 0.2224 (*p_*ρ*_* < 1 × 10^−5^).

As in the case of the data from experimental scenario 2, we control the correlation for the two confounding factors and find that the partial correlation controlled for 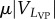 is significant with partial Spearman's rank coefficient equal to *ρ* = 0.1863 (*p_*ρ*_* < 1 × 10^−5^). On the other hand, the correlation disappears when we control it for *μ*|*V*_Ref_| − *μ*|*V*_SF_|. However, because the reference trajectories of the VP were simply made faster by adding 2.5 Hz sine signal, i.e. *μ*|*V*_Ref_| ≥ *μ*|*V*_SH_|, this is exactly what should be expected. In other words, our analysis of the data from the experimental scenario 3 demonstrates the effect of the dynamic similarity in the special case when it can be simplified to a difference between the preferred solo velocities of the participants. It is important to note that this observation is only possible owing to our experimental set-up allowing for an interaction between a human and a VP using as a reference trajectory one of the human player's solo trajectories post-processed as described in the Methods section.

Finally, we control the correlations shown in figure 4 for a multiplicative effect of *μ*|*V*_L_|. In both experimental scenarios, we find that correlation coefficients between mean RPE(*L, F*)/*μ*|*V*_L_| and EMD are statistically significant, *ρ_S_*_2_ = 0.2830 (*p* = 5 × 10^−2^), *ρ_S_*_3_ = 0.1748 (*p* = 2 × 10^−5^). This control further confirms our results.

In summary, we show that a small distance between individual velocity profiles of the leader and the follower, indicating that they have similar movement dynamics, results in higher levels of coordination than those observed in dyads in which the distance between participants' IMS is larger. In so doing, we demonstrate that dynamic similarity affects the level of coordination in joint human movement interactions. Our results are a step forward towards confirming a prediction of the theory of similarity, namely that dynamic similarity enhances interhuman interaction.

## 4. Discussion and conclusion

In this paper, we introduced the notion of dynamic similarity in the mirror game [23] and demonstrated the existence of an IMS in human players. We then showed that the synchronization level between the players is affected by their dynamic similarity. In particular, we proposed the use of velocity profiles, defined by the PDFs of velocity timeseries recorded in the mirror game, as motor signatures. We used the earth's mover distance and MDS to show that velocity profiles of the solo movement have characteristics of IMS, i.e. they are stable over time and differ significantly between individual players. In this way, we revealed time-persistent, individual motor properties that could be detected in complicated, non-periodic motion observed in the mirror game, as suggested in [22]. Notably, we extended the notion of motor signature beyond the frequency content of a periodic motion [17–19,34]. Because the IMS can be readily recorded by means of a cheap off-the-shelf experimental set-up, we believe that it could become an integral part of studies investigating interpersonal interaction.

We introduced the evaluation of the distance between velocity profiles as a method of quantifying dynamic similarity between players' motion in the mirror game and used the RPE to measure their temporal correspondence. Our key finding supports a central prediction of the theory of similarity, specifically that dynamic similarity of participants' solo movements enhances their coordination level [19,35]. A possible mechanisms that could explain the relationship between dynamic similarity and interpersonal coordination might be related to the processes involved in the superior recognition of own motion [36]. In other words, a higher level of coordination between participants with physically similar styles could result from the interplay between motor planning and the visual processing of motion (see [36] and references therein).

Our work complements research on individuality and interactions in animal groups in two ways [37]. First, our study involves direct and intentional coordination that is typical for human–human interactions. Such interactions, in general, allow for investigation of an intentionally designated leader's behaviour and are fundamentally different compared with spontaneous or unintentional coordination amongst individuals and/or groups of animals. Second, the overlap between our results and the findings reported in [37] represents a promising avenue for future work on the extension of animal models to interpersonal interactions.

Finally, the methods we have introduced and the data we have collected establish the use of a VP [38], driven by an ICA, as an effective tool for studying joint actions in the mirror game. Importantly, the advantages of using an ICA based on feedback control to drive the avatar is that bidirectional coupling is maintained during the mirror game and that it allows control of the interaction between human and avatar in two ways, by choosing reference trajectories and by changing the bidirectional coupling parameters of the ICA. Such level of control in a sociomotor coordination task could be used in applications that aim to reinforce social bonding in joint-action tasks [10,39].

In summary

— We introduce quantitative measures and analyse dynamic properties of complex aperiodic movements that characterize human sociomotor interactions.
— We demonstrate the existence of an IMS for each player, defined as a time-invariant, tractable characteristic of her/his movement and reveal that the rapport (similarity) between IMS enhances coordination of movement between different players.
— We introduce a novel ICA able to drive a VP to play the game and employ it as a model of interpersonal interaction in the mirror game between human and VPs.

## Supplementary Material

Supplementary Information

## Figures and Tables

**Figure 5.**
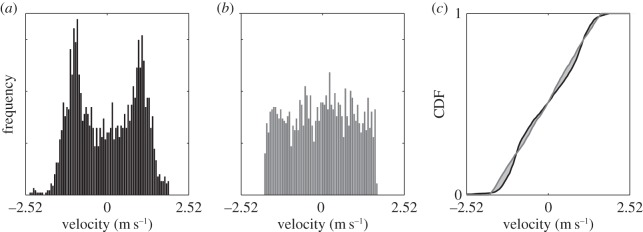
Panel (*a*) shows a velocity profile—histogram (*h_a_*; black) of the velocity timeseries. Panel (*b*) shows histogram (*h_b_*; grey) of a random variable generated with distribution of type 1 from the Pearson system. Both samples have *μ*=−0.01, *σ* = 0.54, *s*=−0.04 and *k* = 1.82, where *μ* is the mean, *σ* is the standard deviation, *s* is the skewness and *k* is the kurtosis. Panel (*c*) shows cumulative density functions of the distribution from panel (*a*) in black and from panel (*b*) in grey. The light grey shading indicates the area of difference between the CDFs, i.e. the EMD between the two distributions, 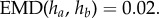

**Figure 6.**
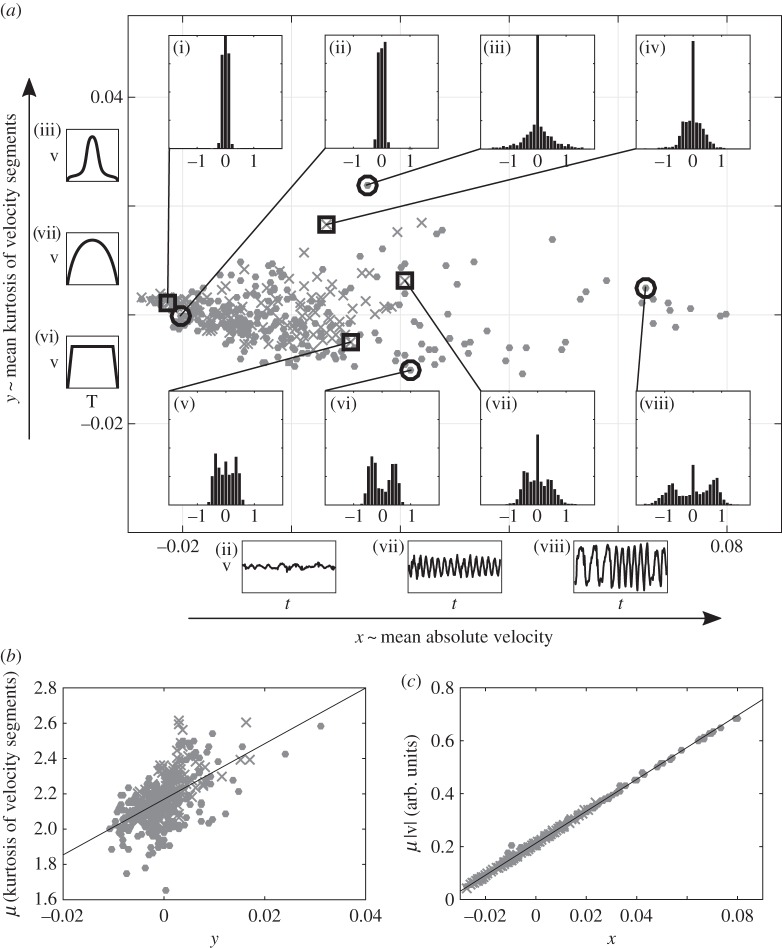
(*a*) *Similarity space* shows points corresponding to velocity profiles of normalized solo data from the scenarios 1 (multiplication symbol) and 3 (bullets). Insets (*a*(i))–(*a*(viii)) show examples of the velocity profiles. *v* is velocity, *t* is time and *T* indicates normalized duration of the velocity segment. (*b*) Relation between *y*-coordinate of the similarity space and average kurtosis of the velocity segments: Corr(*y*, *μ*(kurtosis of vel. segments)): *R*^2^ = 0.5657 (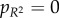), *ρ* = 0.5685 (*p_*ρ*_* = 0). (*c*) Relation between *x*-coordinate of the similarity space and average solo velocity of a participant: Corr(*x*, *μ*|*v*|): *R*^2^ = 0.9993 (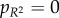), *ρ* = 0.9979 (*p_*ρ*_* = 0).

**Figure 7.**
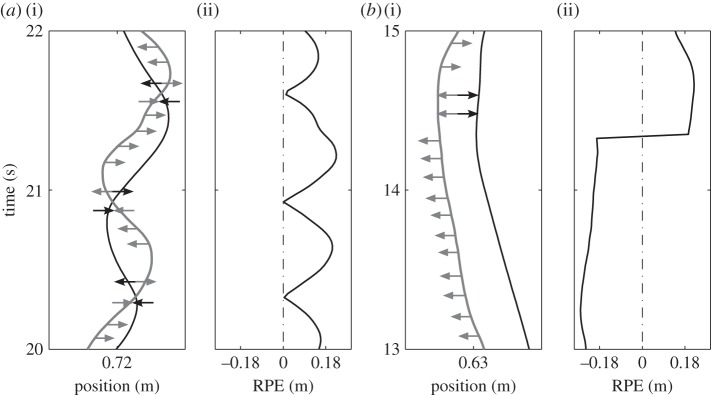
Illustration of the principle used for computing relative position error (RPE). In panels (*a*(i)) and (*b*(i)), the black lines indicate leader's position and the grey lines indicate follower's position. Grey arrows show direction of motion of the follower. At the times indicated by double grey-black arrows, the participants move in opposite directions. Panels (*a*(ii)) and (*b*(ii)) show corresponding RPE (black). Time runs from bottom to top.

## Appendix A. Earth's movers distance

Mathematically, the EMD is a special case of Wasserstein distance [29]. It is a solution of the optimal transportation problem [40] and is an established tool in image analysis and pattern recognition applications [29,30]. For univariate PDFs, it can be expressed in a closed form as the area between their corresponding cumulative distribution functions [41]:

A 1

Here, PDF_1_ and PDF_2_ are two PDFs with support in set *Z*, CDF_1_ and CDF_2_ are their respective cumulative distribution functions. We note that EMD is a well-defined metric in the space of PDFs as it satisfies the following conditions:

These conditions express intuitive notions about the concept of distance. For example, that the distance between distinct points is positive and the distance from *x* to *y* is the same as the distance from *y* to *x*. The triangle inequality means that the distance from *x* to *z* via *y* is at least as great as from *x* to *z* directly. Furthermore, EMD is non-parametric and quantifies partial matches. Hence, it allows to compare PDFs rather than their selected moments. This represents a significant advantage of our analysis in comparison to using selected moments, which are not sufficient to uniquely parametrize a PDF. We note that a bounded PDF is uniquely determined by its moments of all orders (0 to infinity) [42].

Figure 5 shows an example of two histograms with the same estimates of the first four moments and demonstrates that by using the EMD we can distinguish them. The histogram in panel (*a*) is a velocity profile (*h_a_*) computed for a timeseries in our data, whereas panel (*b*) shows a histogram (*h_b_*) of a random variable generated using distribution of type 1 from the Pearson system [43]. Figure 5*c* illustrates how the EMD between two experimental PDFs is computed. The black line in figure 5*c* is the experimental CDF of the histogram from panel (*a*), whereas the grey line is the experimental CDF of the histogram from panel (*b*). The EMD(*h_a_*, *h_b_*) between the two velocity profiles is the area between the black and the grey line, which is indicated with the light grey shading. We note that the maximum of the difference between two CDFs is a basis of the non-parametric goodness-of-fit Kolmogorov–Smirnov test [44].

In practice, to compute empirical CDFs, we take the cumulative sum of histogram bins normalized by the number of data points. We use histograms with 101 equidistant bins over fixed range of velocity values (outliers are assigned to the extreme bins). Furthermore, we normalize the EMDs with the maximal EMD for a given support *Z*; because *|CDF*_1_(*z*) − CDF_2_(*z*) ≤ 1|, the maximal EMD is given by the length of the support EMD_max_ = |*Z*|.

## Appendix B. Similarity space

In figure 6, we illustrate properties of the similarity space. Figure 6*a* shows points corresponding to velocity profiles of solo data from scenario 1 (multiplication symbols) and solo data from scenario 3 (bullets). In order to show the points from two different experimental set-ups in one plot, we normalized position timeseries before computing velocities. Additionally, because the range of motion in the scenario 1 is equal to 60 cm and in the scenario 3, it is equal to 180 cm, we multiplied normalized position data from the scenario 1 by 1/3. Insets (*a*(i)), (*a*(iv)), (*a*(v)) and (*a*(vii)) show examples of velocity profiles from the experimental scenario 1 and (*a*(ii)), (*a*(iii)), (*a*(vi)) and (*a*(viii)) show examples of velocity profiles from the experimental scenario 3.

Further analysis of the data revealed that the *x*-coordinate of the similarity space is correlated with mean absolute velocity *μ*|*V*| (figure 6*c*), and that *y*-coordinate of the similarity space is correlated with mean kurtosis of normalized velocity segments (figure 6*b*), parts of the velocity timeseries between two consecutive points of zero velocity. Mean kurtosis of the velocity segments describes how the participant is changing direction of motion. Low kurtosis indicates sudden changes of directions and relatively constant velocity between them, whereas high kurtosis informs us that the change of direction was slow. The two quantities, taken together, explain accurately general characteristics of the velocity profiles, e.g. bimodality of the velocity profile in the inset (*a*(vi)) means that the participant was moving with a high constant velocity and was quickly changing direction of motion. On the other hand, the velocity profile in inset (*a*(iii)) tells us that the participant was changing direction slowly; the high peak was close to zero velocity and was reaching maximum velocity only for brief moments.

More generally, figure 6 shows that the dimensions of the similarity space, computed using MDS of earth's mover distances between velocity profiles, emerge from properties and characteristics of the human motion in the mirror game. It is important to note that a single property of the velocity profiles, say *μ*|*V*|, is not sufficient to separate different participants. Furthermore, we observe better separation between individuals using the coordinates of the similarity space than using a plane defined by their correlates.

## Appendix C. Relative position error

The RPE is based on the notion that if two objects are moving in the same direction then the one behind is following, and on the assumption that changes in direction of movement are initiated by the leader. We define the RPE as the difference in the players' positions multiplied by their common direction of motion. In cases when the players are moving in opposite directions, we assume that the follower is always behind the leader, regardless of the directions of players' movements. These rules lead to the following rule for computing the RPE(*x*_1_(*t*), *x*_2_(*t*)):

Here, *x*_1_, *v*_1_ are position and velocity of the leader and *x*_2_, *v*_2_ are position and velocity of the follower. Positive values of the RPE mean that the follower is behind the leader. We note that the RPE is not symmetric, that is RPE(*x*_1_(*t*), *x*_2_(*t*)) ≠ −RPE(*x*_2_(*t*), *x*_1_(*t*)) (owing to the assumption that a follower should react to the action of a leader) and therefore, it could be treated as a measure of the performance of the follower in addition to indicating the level of temporal correspondence (coordination).

Figure 7 illustrates the ideas behind computing the RPE. Panels (*a*(i)) and (*b*(i)) depict trajectories of movement of two players, leader position is shown with black line, follower position is shown with grey line; time flows from bottom to top. Figure 7*a*(ii) and *b*(ii) shows corresponding timeseries of the RPE. Grey arrows indicate the direction of motion of the follower. At the times indicated by double grey-black arrows, when the participants move in opposite directions, we compute the RPE using the absolute value of the difference in position, i.e. we assume that the follower is always behind and that the leader initiates changes in the direction of motion.

Figure 7*a* shows how our definition of RPE works for the case when the players are often changing direction of motion. In panel (*a*(ii)), we depict a follower that is always behind (RPE > 0) and that RPE = 0 occurs at times when the players had the same position but were moving in opposite directions. Figure 7*b* shows that if the follower is ahead of the designated leader the RPE is negative. In such a case, we conclude that the designated leader was tracking the movement of the designated follower. Grey-black arrows in figure 7*b*(i) indicate that in the case when the designated leader changed direction of movement, the RPE also changed sign. Comparison of the RPE to other measures of coordination can be found in the electronic supplementary material, §10.
