# Supplementary material for: Dynamic similarity promotes interpersonal coordination in joint action
Source: J R Soc Interface. 2016 Mar;13(116):20151093. doi: 10.1098/rsif.2015.1093 (PMC4843673; doi:10.1098/rsif.2015.1093)
Supplement: Supplementary Information [file rsif20151093supp1.pdf]

Supplementary Information: Dynamic similarity promotes interpersonal coordination in joint-action

Piotr Słowiński[1], Chao Zhai[2], Francesco Alderisio[2], Robin Salesse[3], Mathieu Gueugnon[3], Ludovic Marin[3], Benoit G. Bardy[3,4], Mario di Bernardo[2,5], and Krasimira Tsaneva-Atanasova[1]

[1]Department of Mathematics, College of Engineering, Mathematics and Physical Sciences, University of Exeter, EX4 4QF, United Kingdom
[2]Department of Engineering Mathematics, University of Bristol, Merchant Venturers' Building, BS8 1UB, United Kingdom
[3]EuroMov, Montpellier University, 700 Avenue du Pic Saint-Loup, 34090 Montpellier, France.
[4]Institut Universitaire de France, Paris, France
[5]Department of Electrical Engineering and Information Technology, University of Naples Federico II, 80125 Naples, Italy

1 Preprocessing

- In scenarios 1 and 3, position time series were interpolated with shape-preserving piecewise cubic interpolation (13Hz in experiment 1 and 40Hz in scenario 3).
  Matlab command: `interp1(t,x,0:1/Fs:t(end),'pchip');` Fs is the sampling frequency, t is the series of time and x is the position time series.

- In scenarios 2 and 3, the position data was filtered with a zero-phase forward and reverse digital 2nd order lowpass (10Hz cut-off) Butterworth filter which is a maximally flat magnitude filter.
  Matlab commands: `butter(2,10/(Fs/2))` and `filtfilt`.

- The pre-processed position time series were used to estimate numerically their corresponding velocity time-series. To differentiate position time-series we used a forth-order finite difference scheme. We cut out the first and last 2 seconds of the signal. Furthermore, we limit velocities to 3.5 [a.u./s] in experiment 1 and to 2.7 [m/s] in scenarios 2 and 3 (higher velocities were considered a results of noise in the collected data).

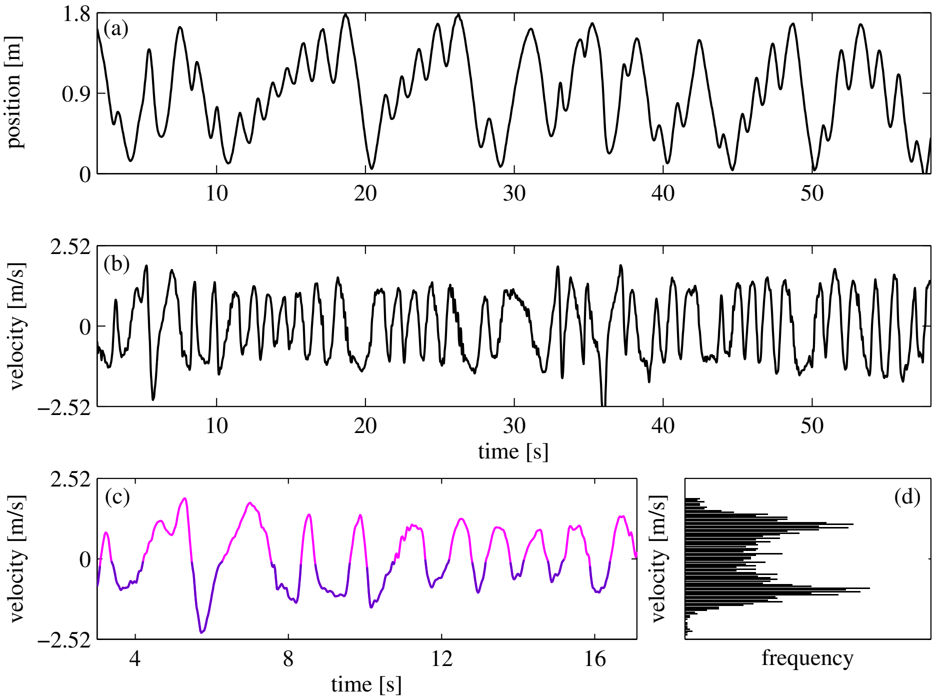

Supplementary Figure 1: (a) An example of solo position time series from the Experiment 3. (b) Velocity estimated from position data. (c) Fragment of the velocity time series with indicated positive velocity segments (magenta) and negative velocity segments (purple). (d) Velocity profile - histogram of the velocity time series.

- To estimate the PDF of the player's velocity we use normalised histogram of the velocity time series with 101 equally distant bins between -2.7 and 2.7 [m/s] (or -3.5 and 3.5 [a.u./s] in Experiment 1).

Supplementary Figure 1 illustrates initial stages of analysis of the data. Supp. Supp. Fig. 1(a) depicts the representative player's position collected in the solo condition in Scenario 3 and (b) its estimated velocity time-series as explained in *Methods*. For each velocity time-series we compute velocity profile, which is the PDF of the player's velocity time series. In Supp. Fig. 1(d) we show the velocity profile of the time series represented in Supp. Fig. 1(b). We use PDFs of velocity in order to capture the essence of the players' movement without being affected by the existing physical constraints on their motion, e.g. limited position range. Multivariate distributions, involving consideration of more than one feature of the motion, would describe the dynamics in a more detailed way but we found that univariate distributions, namely of player's velocities [1, 2, 3, 4, 5, 6, 7] contain enough information to achieve the goals of our study.

Finally, Supp. Fig. 1(c) depicts the first 20 seconds of the time series from Supp. Fig. 1(b), with indicated positive velocity segments (magenta) for velocities bigger than 0 that correspond to the movements of the hand from "left to right", and negative velocity segments (purple) with velocities smaller than 0 that correspond to movements from "right to left". To estimate the PDF of the player's velocity we use normalised histogram of the velocity time series with 101 equally distant bins between -2.7 and 2.7 [m/s] (or -3.5 and 3.5 [a.u./s] in Experiment 1). In Supp. Fig. 1(d) we show the velocity profile of the time series represented in Supp. Fig. 1(b). In order to compare velocity profiles with velocity segments, for each velocity time-series from the experiments 1 and 3 we also find their velocity segments. Velocity segments are fragments of the velocity time series between two consecutive points of zero velocity, i.e. each velocity segments corresponds to a short movement in one direction. For our analysis, we normalise the velocity segments and compute their curve moments. Following [1, 6] we take into account only velocity segments that are longer than 0.2 sec., shorter than 8 sec. and which have displacement larger than 0.03 [m] (before normalisation). Note that, for the velocity segments, the moments of curve are computed with respect to time and hence parametrise the shape of the velocity segments rather than moments of the sample of velocity (see Section 4 of SI).

2 Earth's movers distance

EMD can be computed using the following Matlab code:

```
bins=linspace(z1,z2,101);    % support Z with 101 bins
bin_width=bins(2)-bins(1);   % widths of the bins, i.e. dz
max_emd=abs(z2-z1);          % maximal EMD

h1=hist(v1,bins);    % h1 and h2 are velocity profiles
h2=hist(v2,bins);    % v1 and v2 are velocity time series
l1=numel(v1);        % for normalistion
l2=numel(v2);        % for normalistion
emd_v1v2=sum(abs(cumsum(h1/l1)-cumsum(h2/l2)))*bin_width/max_emd;
```

3 Multidimensional scaling

We use multidimensional scaling (MDS) to study relations between players' velocity profiles. MDS allows us to model the players' motion as points in an abstract geometric space, which we shall refer to as *similarity space*. It is a well established tool in data visualisation and data mining [13]. It allows to reduce dimensionality of the data and visualise relations between the objects

under investigation while preserving as much information as possible. Since the EMD is a metric in the space of velocity profiles (defined by the PDFs of velocity time series), we use classical MDS as implemented in Matlab. We use the Matlab command: `cmdscale`.

In particular, we first compute the EMDs between all the analysed PDFs, which correspond to individuals' movements. Then we use the computed EMDs in order to construct a matrix $\mathbf{D}$. Each row of this matrix is assigned to a different PDF (and hence belongs to a specific individual, i.e. participant in the mirror game) and contains EMDs between this PDF and all the other PDFs. For instance, cell (2,3) contains the EMD between second and third PDFs in our dataset. Since the EMD is a metric, matrix $\mathbf{D}$ has zeros on the diagonal and is symmetric.

Next, we use the MDS to transform matrix $\mathbf{D}$ into coordinates of points in the *similarity space*. In this way each velocity profile is represented as a single point in the *similarity space*. Here we use only the first two dimensions of the *similarity space*, which were found to be sufficient for the purpose of our analysis. These two dimensions correspond to the first two highest eigenvalues of matrix $\mathbf{D}$ computed in the MDS.

The MDS algorithm is implemented as follows:

1. Take $n \times n$ matrix $\mathbf{D}$ (n number of analysed objects), and square its elements in order to obtain matrix $\mathbf{D}^2$.

2. Transform matrix $\mathbf{D}^2$ into matrix $\mathbf{B}$; subtract row means, subtract column means, add back (grand) mean of all the matrix elements and multiply by -0.5. Formally this operation is called double centring and can be expressed as: $\mathbf{B} = -0.5\mathbf{J}\mathbf{D}^2\mathbf{J}$, here $\mathbf{J} = \mathbf{I} - 1/n\mathbb{1}\mathbb{1}^T$, where $\mathbf{I}$ is the identity matrix, $\mathbb{1}$ is the vector of ones of length n, and $\mathbb{1}^T$ is the transposed vector $\mathbb{1}$.

3. Factor $\mathbf{B}$ by its eigendecomposition $\mathbf{B} = \mathsf{E}\Lambda\mathsf{E}^T$, where E is matrix which has eigenvectors of $\mathbf{B}$ as columns, and Λ is a diagonal matrix with ordered eigenvalues $\lambda_1 \geq \lambda_2 \geq ... \geq \lambda_n$ on the diagonal.

4. Take the first m eigenvectors E_m and eigenvalues Λ_m of matrix $\mathbf{B}$ and compute $\mathbf{X} = \mathsf{E}_m\Lambda_m^{1/2}$; $\mathbf{X}$ is a $n \times m$ matrix with m coordinates for each of the n analysed objects; the MDS relies on the property that the eigenvectors of matrix $\mathbf{B} = \mathbf{X}\mathbf{X}^T$ can be interpreted as geometric coordinates; $\mathbf{X}^T$ is transposed matrix $\mathbf{X}$.

MDS is a technique related to principal component analysis (PCA) [13]. In particular, PCA is a statistical procedure which uses singular value decomposition of a matrix $\mathbf{Y}$ or eigendecompostion of covariance matrix $\mathbf{Y}^T\mathbf{Y}/(n-1)$ to study underlying structure of the data. Here $\mathbf{Y}$ is a centred (i.e. its columns have removed means) $n \times m$ matrix of n observation vectors $\tilde{Y}$. In other words, the PCA uses the eigenvectors of the covariance

matrix to perform orthonormal transformation of the original coordinate system of the data, i.e. it projects the data into an abstract geometric space with dimensions given by linear combinations of the the original variables. In the same way, the MDS uses eigenvectors and eigenvalues of matrix **B** to find a geometric model of the data in an abstract geometric space. The difference between PCA and MDS is the origin and nature of the decomposed matrix. The results of both procedures are eigenvectors and eigenvalues which can be used to reduce the dimensionality of the data while preserving covariance of data (PCA) or preserving distances between analysed objects (MDS). In the case in which distances between analysed objects are given by covariances, i.e. $\mathbf{D} = 1 - \mathbf{Y}^T\mathbf{Y}$, or if $\mathbf{D}$ is given by euclidian distances between n observations vectors $\tilde{Y}$, both methods give the same results.

The MDS allows us to extend pair-wise analysis of distances between velocity profiles and gain further insight into our data [14]. Furthermore, using MDS guarantees that the euclidian distances between elements in the *similarity space* are a good approximation of the EMDs between velocity profiles. Since EMD is a metric we know that the dynamic similarity between players' movements is reflected in the Euclidean distances between their respective positions in the *similarity space*, i.e. the closer the points in *similarity space*, the more similar their velocity profiles. This renders the *similarity space* a key tool in our analysis of how the dynamic similarity affects mutual rapport and coordination between players in the mirror game.

4 Moments of a curve

Supplementary Table 1: Definitions of the first 4 moments of function (curve) $f(t)$ with support/ defined on T=$[t_1, t_2]$.

1st moment — centre of mass	$\mu = \int_{t_1}^{t_2} t f(t) dt$
2nd moment — variance	$\sigma = \int_{t_1}^{t_2} (t-\mu)^2 f(t) dt$
3rd moment — skewness	$s = \frac{1}{\sigma^{\frac{3}{2}}} \int_{t_1}^{t_2} (t-\mu)^3 f(t) dt$
4th moment — kurtosis	$k = \frac{1}{\sigma^2} \int_{t_1}^{t_2} (t-\mu)^4 f(t) dt$

To analyse and compare movements of different participants Noy et. al. use skewness and kurtosis of normalised velocity segments [1, 6]. To perform a meaningful comparison of the moments of different functions $f(t)$, it is necessary to rescale their supports T to a common one, and to normalise

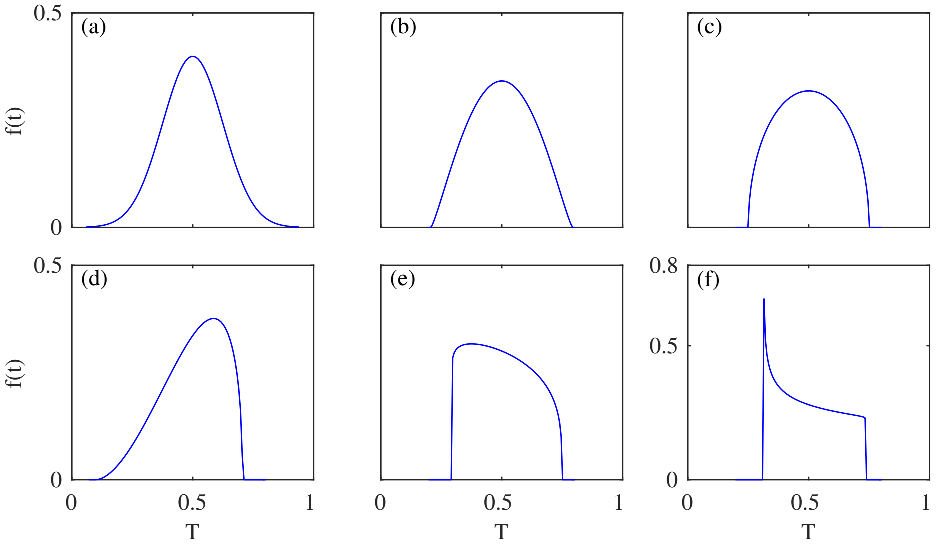

Supplementary Figure 2: Different curve segments (a) Normal distribution $s = 0, k = 3$; (b) Minimum jerk $s = 0, k = 2.2$; (c) $s = 0, k = 2$; (d) $s = -0.5, k = 2.5$; (e) $s = 0.15, k = 1.9$; (f) $s = 0.2, k = 1.8$. For all distributions: $\mu = 0.5, \sigma = 1$.

the functions $f(t)$ with their integrals $\int_{t_1}^{t_2} f(t)dt$. In particular, in [6] as well as in our analysis the support T of the velocity segments is time normalised into $\tau \in [0, 1]$. We note that moments of curve are different from moments of a sample, i. e. they are computed with respect to the support, rather that the values in the sample. For example, in the case of the centre of mass, the area under the curve on the left side of the centre of mass μ is equal to the area under the curve on the right side of it, that is μ, $\int_{t_1}^{\mu} f(t)dt = \int_{\mu}^{t_2} f(t)dt$.

Supplementary Figure 2 depicts six examples of normalised curve segments with support $T \in [0, 1]$; all presented functions have the same centre of mass $\mu = 0.5$ and variance $\sigma = 1$, whilst skewness and kurtosis vary between panels. In the case of a velocity segment, skewness indicates asymmetry in acceleration and deceleration, while kurtosis provides information about uniformity of the maximal velocity. Low kurtosis means that an object is quickly accelerating and decelerating and keeps constant velocity in between while high kurtosis means that the object is accelerating slowly, and after reaching maximum velocity it almost immediately starts to slow down; normalised velocity segments with higher kurtosis, generally, have higher maximum velocity.

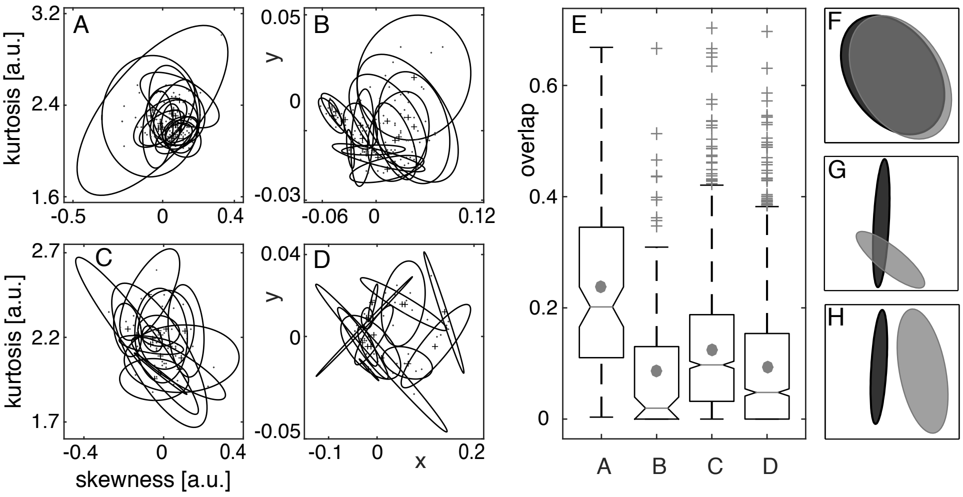

Supplementary Figure 3: Individual motor signature in A the kurtosis-skewness of velocity segments plane [1] and in B the *similarity space* computed with MDS from distances between velocity profiles. For 15 different participants from experimental Scenario 1, on three different days with at least one week break between recording rounds. Positive velocity segments and velocity profiles from 56 solo trials of 14 participants from experimental Scenario 3 shown in C the skewness-kurtosis plane and in D the *similarity space* (for the sake of clarity data for only 14 out of 51 participants is shown). Each ellipse corresponds to a different participant. Small dots correspond to individual solo recordings. Each cross at the centre of an ellipse corresponds to the average of the small dots' positions. Each ellipse indicates 0.7 mass of bivariate normal distribution fitted to the small dots (see *SI appendix* for further details). Box plots in panel E show distributions of overlaps ω between pair of ellipses in panels A–D. Line between notches indicates median, dot indicates mean. The "central box" represents the central 50% of the data and its lower and upper boundary lines are at the 25%/75% quantile of the data. The two vertical lines extending from the central box indicate the remaining data outside the central box that are not regarded as outliers, crosses indicate outliers. Panels F–G show examples of overlap ω between pair of ellipses: F the ellipses almost completely overlap, ω=0.91; G the ellipses partially overlap, ω=0.13; H the ellipses do not overlap, ω=0.

5 Comparison of velocity segments and velocity profiles

In this section we compare two candidates for the individual motor signature. Following [1] we begin our analysis by studying kurtosis and skewness of velocity segments. Velocity segments, which were used to analyse the mirror game in [1, 6], are parts of the velocity time series where the participant

is moving in one direction, i.e., parts of the velocity time-series between two consecutive times of zero velocity. From the viewpoint of motion dynamics, skewness indicates asymmetry in acceleration and deceleration in a velocity segment, while kurtosis provides information about uniformity of the maximal velocity in a velocity segment. Low kurtosis indicates that an object was quickly accelerating and decelerating, and kept maximal velocity for a long time. High kurtosis, on the contrary, means that the object was accelerating slowly, and moved with maximal velocity only for a short period of time.

5.1 Overlap

In order to analyse separation and clustering of data points corresponding to velocity profiles of individual participants in the *similarity space* or on the plane of skewness and kurtosis of velocity segments, we encircle them with ellipses given by bivariate gaussian distribution fitted to their coordinates, and next we compute how much the ellipses overlap. In practice we, first, compute mean values and covariance matrix of coordinates of the n points which we wish to encircle. The points correspond to n trials of a subject. Mean values of the coordinates give the position of the centre of the ellipse, while the eigenvectors of the covariance matrix give directions of major and minor axes of the ellipse. Finally, the lengths of the axes of a covariance ellipse that encloses the desired probability mass are given by the square roots of the eigenvalues of the covariance matrix multiplied by the Mahalanobis radius [15]. In our analysis we use a radius that encloses all of the data points of a participant, which corresponds to 0.7 of the probability mass of the bivariate normal distribution.

We compute the overlap, ω, between ellipses as a ratio of the area of intersection and the total area of two ellipses. In this way total separation corresponds to no overlap $\omega = 0$, whilst complete overlap $\omega = 1$ means that we cannot distinguish between the two ellipses and hence we cannot distinguish between points that are encircled by them. The overlap ω between ellipses allows to assess clustering and separation between regions of the *similarity space*, or the plane of skewness and kurtosis of velocity segments, corresponding to different individuals. The advantage of this simple method is that it is dimension independent and hence allows to compare clustering and overlap in different spaces.

5.2 Comparison

Supplementary Figure 3B depicts velocity profiles of individual players presented as elements of the *similarity space*. Supplementary Figure 3E clearly demonstrates that the median overlap $\tilde{\omega}$ between ellipses, and hence individuals, in the skewness-kurtosis plane $\tilde{\omega}_A = 0.2$ is much higher than the

median overlap between ellipses in the *similarity space* $\tilde{\omega}_B = 0.02$ (significance $p_{AB} < 0.0001$, Kolmogorov-Smirnov test [**?**, 16]). More importantly, there are 45 out of 105 pairs of ellipses that do not overlap at all in the *similarity space* while in the kurtosis-skewness plane all pairs of ellipses overlap, ($\min \omega_A = 0.004$). To verify our results we also analyse solo recordings collected in the experimental Scenario 3. In the experimental Scenario 3, players had larger range of movement and all the solo trials of individual players were recorded on a single day. Nevertheless in this case we also find that the median overlap $\tilde{\omega}$ between ellipses in the skewness-kurtosis plane $\tilde{\omega}_C = 0.1$ is much higher than the median overlap between ellipses in the *similarity space* $\tilde{\omega}_D = 0.05$ (significance $p_{CD} < 0.0001$) and the number of non-overlapping pairs of ellipses is higher in the *similarity space* (418 against 183 out of 1378 pairs); see Supp. Fig. 3C and D. In both experimental scenarios, we observe that separation between ellipses, and hence individuals, is significantly better in the *similarity space.*

Our analysis reveals that, despite being a good source of information about human movement on a short time-scale (rates of acceleration, uniformity of maximal velocity), velocity segments are not specific enough to study the effects of dynamic similarity between individual players. More specifically, we find that although the mean values of kurtosis and skewness of velocity segments exhibit clustering for each person, and that the clusters are preserved over time, there also exists a big overlap between mean skewness and kurtosis of different players, meaning that it is not possible to distinguish between them; see Supp. Fig. 3A, C and E.

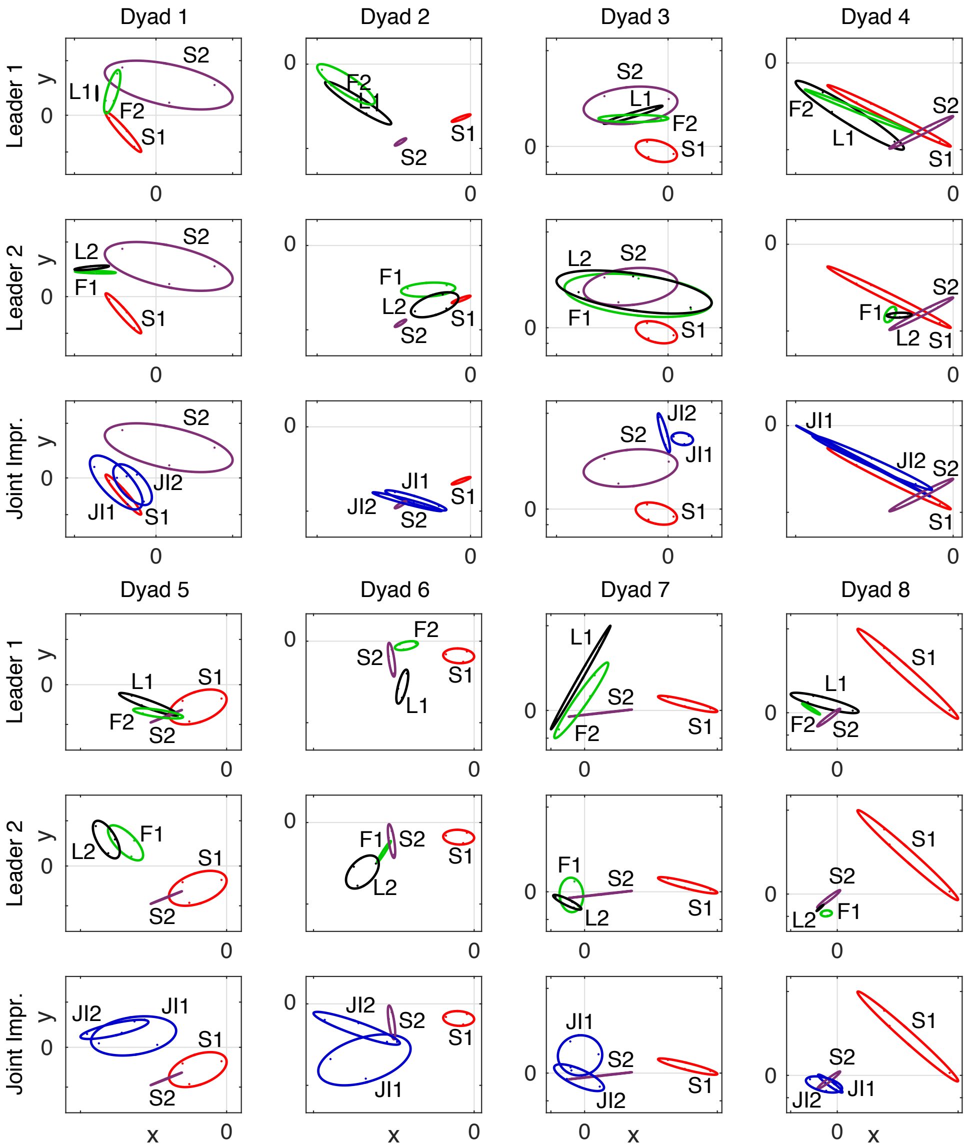

Supplementary Figure 4: Interaction between two players in different experimental conditions (Scenario 2) visualised in the *similarity space* for all 8 dyads. Ellipses encircle points corresponding to velocity profiles in solo (S1 and S2; red), leader (L1 and L2; black), follower (F1 and F2; green) and joint improvisation (JI1 and JI2; blue) rounds. Each column depicts data for two different dyads. x and y axis are rescaled for clarity of presentation.

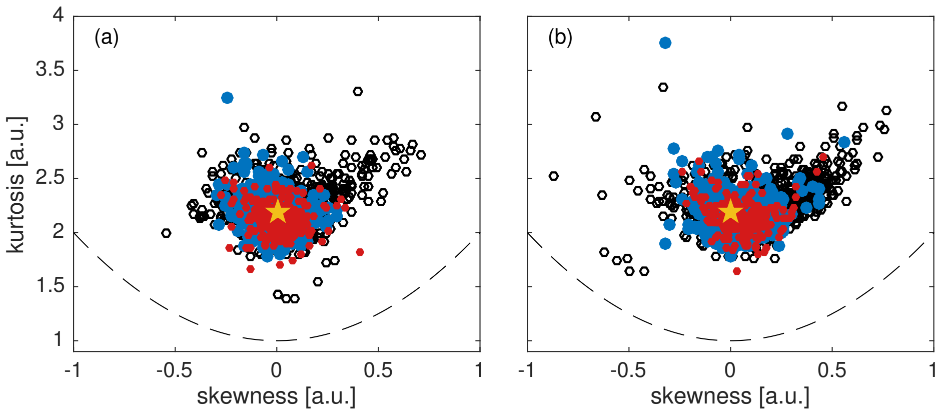

Supplementary Figure 5: Kurtosis and skewness for velocity segments from time series recorded in the experiment. In panel (a) values from positive velocity segments, in (b) from negative velocity segments; compare with Supp. Fig. 1(c). In black skewness and kurtosis of velocity segments from ICA acting as a leader. In blue skewness and kurtosis of velocity segments from human players acting as a leader. In red skewness and kurtosis of velocity segments from human players in solo conditions. Yellow star indicates point with $k = 2.2$ and $s = 0$ which corresponds to the smoothest movement [1]. Dash-dotted line shows theoretical bound given by relation between kurtosis and skewness of a curve $k \leq s^2 + 1$ [30].

6 Velocity segments of movement generated by ICA

In this section we use kurtosis and skewness of velocity segments to show that the trajectories generated by the ICA [28, 29] in Scenario 3, have the features of a human movement. In our analysis we compared skewness and kurtosis of: velocity segments of human solo movement, velocity segments of human leader movements, and velocity segments of motion generated by the ICA as a leader. We found that except for few outliers, the velocity segments generated by the ICA have the same kurtosis and skewness as the one observed in human motion.

Supplementary Figure 5 shows kurtosis and skewness of velocity segments from time series recorded in experiment 3, (a) for positive velocity segments and (b) for negative velocity segments. Corresponding to the movement of the hand towards and away from the centre of the body, respectively. The skewness and kurtosis of velocity segments from human solo is depicted in red, human leader in blue and avatar leader in black. We notice that for some points the skewness of the avatar leader (black) have bigger values than typical human leader (blue), this is due to the fact that, while

leading the human follower, the avatar was at the same time tracking the fast changing pre-recorded reference trajectory. In other words, the stronger skewness indicates that the avatar was quickly accelerating to match the pre-recorded position and then slowly decelerating to allow human follower to catch up. Nevertheless, overall the black dots and blue circles occupy a similar region of the kurtosis-skewness plane. In both panels all the velocity segments are centred on the point with skewness $s = 0$, and kurtosis $k = 2.2$, which is indicated by a cyan star. This point corresponds to the velocity segment with the smoothest movement as reported in [1, 6]. Normal distribution has skewness $s = 0$, and kurtosis $k = 3$. Dash-dotted line shows theoretical bound of the values of skewness and kurtosis given by the theoretical relation between them $k \leq s^2 + 1$ [30].

Supplementary Table 2: Skewness of positive s_+ and negative s_- velocity segments from different movement recordings: S - human solo movement, L_h - human leader, F_a - avatar follower, L_a - avatar leader, F_h - human follower. p_{ks} is significance level of Kolmogorov-Smirnov test.

	s_+	s_-	p-value
S	0.0098	0.0489	p_{ks}=9.5e-4
L_h	-0.0070	0.0291	p_{ks} <0.0001
L_a	0.0473	0.1320	p_{ks} <0.0001
F_h	0.0913	0.0658	p_{ks}=7.8e-4

In Supplementary Table 2 we report the difference in skewness of the positive and negative velocity segments. This difference is a result of asymmetry in the movement of the hand towards and away from the centre of the body, i.e., the movement is actuated by different groups of muscles [31]. We have not found difference between kurtosis of positive and negative velocity segments in any condition.

In summary, Supp. Fig. 5 shows that in most trials, velocity segments of the avatar leader (driven by ICA) have kurtosis and skewness which are very similar to the human players. Furthermore, higher than in Solo condition values of skewness of the human follower's velocity segments are consistent with the observation that after noticing changes of direction of the leader's movement, the human follower reacts and accelerates quickly to correct her/his position. Next, she/ he slows down to track the leader's movement in a more precise way.

Supplementary Table 3: Partial correlations between RPE and EMD controlled for $\mu|V_L|$, and RPE and average absolute velocity of the leader $\mu|V_L|$ controlled for EMD in data from scenario 2 and scenario 3.

Scenario 2:	$\mu\|V_L\|$	$EMD(S_1, S_2)$
$RPE(L, F)$	ρ=0.3448 (p_ρ=0.0189)	ρ=0.3466 (p_ρ=0.0183)

Scenario 3:	$\mu\|V_{LVP}\|$	$EMD(Ref, S)$
$RPE(L_{VP}, F_H)$	ρ=0.6554 (p_ρ=0)	ρ=0.1863 (p_ρ=0.4e-05)

7 Correlations and partial correlations between of different variables and RPE

Considering the characteristics of motion, the effect of adding a 2.5Hz sinusoidal signal to the solo trajectories results in higher average absolute velocity of the reference trajectory: $R^2(\mu EMD(Ref, S), \mu|V_{Ref}| - \mu|V_S|)$=0.9676 (p = 0), Spearman's $\rho(\mu EMD(Ref, S), \mu|V_{Ref}| - \mu|V_S|)$=0.9689 (p = 0). Nevertheless, we still found significant effect of the dynamic similarity controlled for average absolute velocity of the leader, see Supplemetary table 3, Scenario 3. The existence of this correlation confirms that the velocity of the leader's motion alone could no explain the variability in the RPE, even in the very limited case when dynamic similarity simplifies to differences between preferred (solo) velocities of the players.

8 Statistical tests

Since our data is not normally distributed, to test the existence of correlations we use Spearman's rank correlation coefficient ρ [16]. Additionally, for illustrative purposes, we compute the Pearson R^2 coefficient of linear dependance. To compute correlation coefficients and their significance values (p-values) we use Matlab commands:
`[R2,p]=corr(x,y,'type','Pearson')` and
`[rho,p]=corr(x,y,'type','Spearman')`.
For the same reason, to test statistical significance of differences between distributions we use Kolmogorov-Smirnov test computed with Matlab command `kstest2`. Kolmogorov-Smirnov test determines whether independent random samples are drawn from the same underlying continuous population [16].

Supplementary Table 4: Correlations between RPE and different measures of similarity between solo recordings. Relation between data is measured with Pearson R^2 and Spearman's rank correlation coefficient ρ. $\mu|V_{S\cdot}|$ is average absolute solo velocity, $\max|V_{S\cdot}|$ is maximum absolute solo velocity, $sk_{S\cdot}$ is mean skewness of the solo velocity segments, $kr_{S\cdot}$ is mean kurtosis of the solo velocity segments, $(sk, kr)_{S\cdot}$ are the coordinates of a centre of player's ellipse in the skewness-kurtosis plane obtained from the solo trials.

	$\mu RPE(L,F)$	$\mu RPE(L_{VP}, F_H)$
$EMD(S_L, S_F)$	R^2=0.3701<br>(p_{R^2}=0.0105)<br>ρ=0.3907<br>(p_ρ=0.0066)	R^2=0.2343<br>(p_{R^2}=4.5e-09)<br>ρ=0.2224<br>(p_ρ=2.7e-08)
$\text{abs}(\mu\|V_{SL}\| - \mu\|V_{SF}\|)$	R^2=0.3469<br>(p_{R^2}=0.0169)<br>ρ=0.3453<br>(p_ρ=0.0175)	R^2=0.3281<br>(p_{R^2}=8.0e-17)<br>ρ=0.2997<br>(p_ρ=3.6e-14)
$\text{abs}(\max\|V_{SL}\| - \max\|V_{SF}\|)$	R^2=0.1110<br>(p_{R^2}=0.4576)<br>ρ=0.1383<br>(p_ρ=0.3538)	R^2=0.1154<br>(p_{R^2}=0.0043)<br>ρ=0.0824<br>(p_ρ=0.0415)
$\text{abs}(sk_{SL} - sk_{SF})$	R^2=0.3834<br>(p_{R^2}=0.0078)<br>ρ=0.3152<br>(p_ρ=0.0309)	R^2=0.2124<br>(p_{R^2}=1.1e-07)<br>ρ=0.1427<br>(p_ρ=4.0e-04)
$\text{abs}(kr_{SL} - kr_{SF})$	R^2=0.0686<br>(p_{R^2}=0.6468)<br>ρ=0.1405<br>(p_ρ=0.3461)	R^2=-0.2271<br>(p_{R^2}=1.3e-08)<br>ρ=-0.2287<br>(p_ρ=1.0e-08)
$\|\|(sk,kr)_{SL} - (sk,kr)_{SF}\|\|_2$	R^2=0.1058<br>(p_{R^2}=0.4791)<br>ρ=0.2132<br>(p_ρ=0.1501)	R^2=-0.1346<br>(p_{R^2}=8.4e-04)<br>ρ=-0.0835<br>(p_ρ=0.0389)

9 Relative phase

Analysis of the relative phase between two (or more) oscillators is an established method for quantifying synchronisation (coordination) level and hence temporal correspondence between periodic time series [17, 18]. We performed such analysis by using a method of reconstructing phase of an oscillator from data as described in [19, 20]. In particular, following [19], we computed protophase using the Hilbert transform and transformed it into phase, which grows linearly with time, using the Damoco 2 toolbox for Matlab [21]. However, measures of temporal correspondence that rely on the relative phase based on the Hilbert transform are not suited for the analysis of the time series recorded in our experiments for the following reasons:

- they have non-zero local mean; signal with moving averages have big jumps in phases which introduce big errors in relative phase (see Fig. 2 in [17]),
- their amplitude and phase spectra are not well separated; relative phase is sensitive to changes of amplitude,
- in many cases the time series contain multiple frequencies; instantaneous phase based on the Hilbert transform can be computed but does not have a physical interpretation.

More information about issues stated in the above list and importance of different assumptions for correct estimation of the phase of a signal can be found in [17].

Since the results obtained from the analysis of relative phase based on Hilbert transform were not satisfactory, we decided to use a method of estimating the relative phase based on a wavelet transform of a time series [22, 23, 24]. In particular, we used estimation of relative phase based on wavelet coherence as described in [25] and implemented in Crosswavelet and Wavelet Coherence toolbox for Matlab [26]. Wavelet coherence can be considered a localised correlation coefficient in time-frequency space.

Wavelet coherence provided us with an estimate of relative phase in the time-frequency space, i.e. at each time we have multiple values of relative phase that correspond to different frequencies. To reduce dimensionality of the time frequency estimate of the relative phase, we averaged it over frequencies, obtaining in this way the time course of relative phase.

Supplementary Figure 6 illustrates the process of averaging the estimate of the relative phase computed in the time-frequency space over frequency. In particular, Supp. Fig. 6(a) shows the position time series of leader (black) and follower (green), while Supp. Fig. 6(b1) shows the values of the wavelet coherence in the time-frequency space. Red colours indicate high coherence, i.e. the two signals have correlated frequency components at a given time,

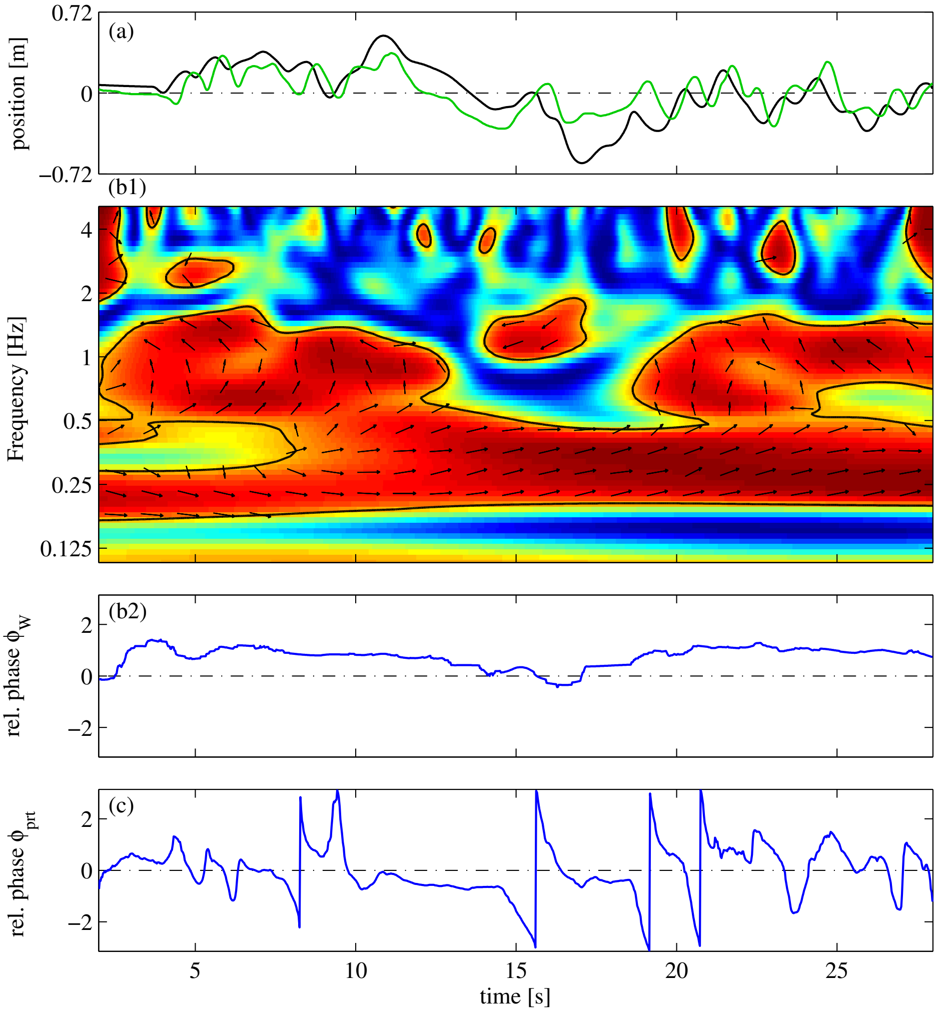

Supplementary Figure 6: Relation between wavelet coherence and relative phase. (a) Position time series of leader (black) and follower (green). (b1) Squared wavelet coherence between leader and follower time series, red indicates high coherence and blue low coherence. Black contour shows 0.05 significance level, arrows indicate relative phase relationship (clockwise positive values, anti-clockwise negative values). (b2) Relative phase computed with significant wavelet coherence averaged over frequencies. (c) Relative phase between time series based on Hilbert transform.

whilst blue ones indicate regions with no coherence. Black contour delineates the area where common frequencies of both signals are statistically significant; tested against random noise [25]. Arrows are a visualisation

of the phase relation between correlated frequency components of the two time series (clockwise angles have negative values, anti-clockwise angles have positive values). Arrows pointing to the right show that the two signals are in-phase. For clarity only arrows in the regions with statistically significant coherence are shown.

Supp. Fig. 6(b2) shows frequency average of relative phase ϕ_W from the regions with statistically significant coherence; we use circular mean to compute the average [27]. Supp. Fig. 6(c) shows the relative phase based on the Hilbert transform ϕ_{prt} computed with the Damoco 2 toolbox [26]; multiple jumps in the relative phase are caused by the changes in the local means of the two signals. This figure clearly demonstrates that the estimate of the relative phase computed with wavelet coherence ϕ_W is better than the one based on Hilbert transform ϕ_{prt}, since the sign of the former is consistent with the fact that the designated leader was actually leading the other player during the joint action. The advantage of this method originates from the fact that ϕ_W is based on the parts of the signal which are measurably correlated and can be modelled with periodic functions in the time-frequency plane.

10 Comparison of different measures of temporal correspondence

Having introduced different measures of temporal correspondence in the sections above we now compare the relative error in position and the estimate of the relative phase based on wavelet coherence, using data from experiment 3 (we used data from experiment 3 because it contains the largest number of trials).

Supplementary Figure 7(a) shows position time series of leader (black) and follower (green) (the plots do not start from zero because we cut out the first 2 seconds of the signals). Supp. Fig. 7(b) shows the RPE between the positions in panel (a), while Supp. Fig. 7(c) shows the estimate of relative phase based on the wavelets coherence. We observe in Supp. Fig. 7(b) and (c) that the RPE behaves differently compared to the relative phase, e.g. in the time interval [10,15] the RPE indicates that the follower is ahead of the leader ($RPE < 0$), while the relative phase shows that there was no exchange of roles between leader and follower. This different behaviour is caused by the fact that the RPE is computed using the information at a given instant of time, while the estimate of the relative phase is based on the wavelet transformation for which time localisation depends on the frequency and is limited by the time-frequency uncertainty principle [25]. Also, in this time interval there are no fast oscillations in the movement, therefore the phase was estimated using low frequency wavelets for which the relative phase was positive (compare with Supp. Fig. 6(b)).

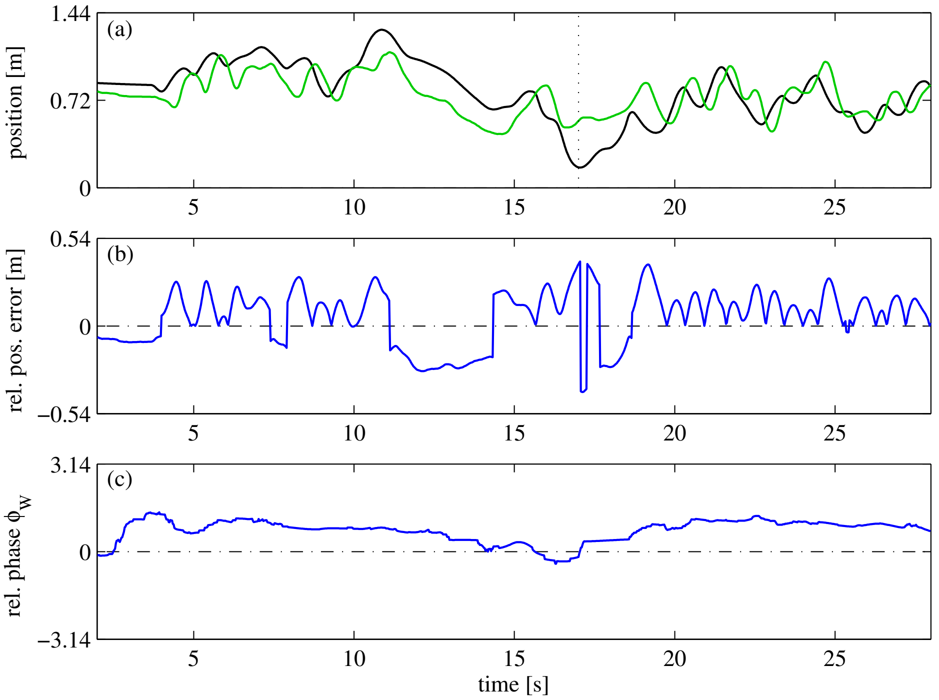

Supplementary Figure 7: Comparison of relative position error and relative phase computed with wavelet coherence. (a) position time series of leader (black) and follower (green). (b) Relative position error. (c) Relative phase computed with wavelet coherence ϕ_W.

The negative value of the relative phase in Supp. Fig. 7(c) around $t = 17s$ is caused by the temporal mismatch between the minimum in the follower's trajectory (green) and the next minimum on the leader's trajectory (black). The minimum in the leader's trajectory indicated by the vertical dotted line in panel (a) occurs after the green one, while all the other extrema of the black trace precede the green trace extrema. The relatively fast change in trajectory in this case was estimated by using higher frequency wavelet of short duration for which the relative phase was negative. Observations from Supp. Fig. 7(c) are consistent with the RPE in panel (b) which has negative values for a short time around $t = 17s$.

More generally, based on our analysis and the example discussed above, we conclude that in the context of the mirror game, where the players move along complicated trajectories, the most useful method for quantification and assessment of the leader-follower interaction is the RPE measure. Specifically, the RPE exhibits stronger association to the dynamics of the movement (in terms of statistical significance of the results of the analysis), than the relative phase. Nevertheless, we envisage that the relative phase

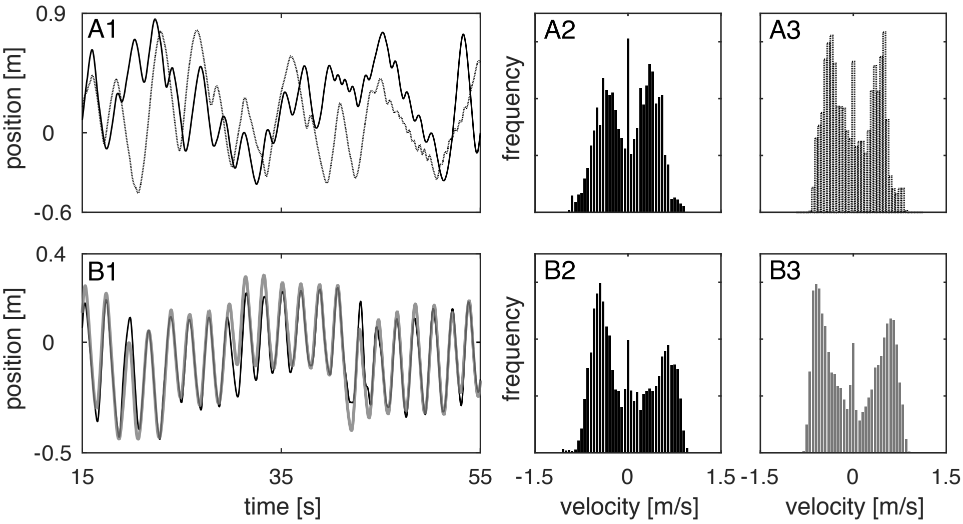

Supplementary Figure 8: Top row shows an example of nontrivial dynamic similarity: panel A1 shows two solo movement trajectories of the same participant, corresponding velocity profiles are shown in panels A2 and A3; RPE_{A1}=0.19, $EMD(h_{A2}, h_{A3})$=0.009. Bottom row shows an example of trivial dynamic similarity: panel B1 shows movement trajectories of a leader (black) and a follower (grey), corresponding velocity profiles are shown in panels B2 and B3; RPE_{B1}=0.008, $EMD(h_{B2}, h_{B3})$=0.008.

based on wavelet coherence would be useful when analysing data from the mirror game played in a condition without designated leader, e.g. joint improvisation, when the RPE cannot be used. Finally, an advantage of using the RPE to quantify temporal correspondence is its straightforward physical interpretation.

11 On the relation between temporal correspondence and dynamic similarity

Given two complex time-series, regardless of their origin, it is always possible to measure their temporal correspondence, e.g. using relative position error (RPE), as well as compute their dynamic similarity, using earth's mover distance (EMD) between histograms of their first derivative. By comparing these two quantities, we can define trivial and nontrivial dynamic similarity.

More specifically, if the two position time series are coordinated, they will necessarily have similar velocity profiles. For example, consider perfect synchronisation when two time series are identical, in such case also their velocity profiles have to be identical and the EMD between them equals 0, i.e. good coordination ⇒ small EMD. Dynamic similarity, which is a result

of the synchronisation between time series, shall be called trivial. On the other hand, if the EMD between velocity profiles is small, while position time series are uncoordinated we observe nontrivial dynamic similarity i.e. small EMD $\not\Rightarrow$ high coordination. Such situation is possible because velocity profiles do not contain temporal information.

Supplementary Figure 8 illustrates the difference between trivial and nontrivial dynamic similarity. In panel A we depict two trajectories of solo movement of a player and their corresponding velocity profiles. The mean RPE between the two trajectories in panel A1 is equal to $\mu RPE_{A1} = 0.19$, and the EMD between histograms in panels A2 and A3 is equal $EMD(h_{A2}, h_{A3}) = 0.009$; it is an example of nontrivial dynamic similarity. In panel B we depict leader (black) and follower (grey) trajectories and their corresponding velocity profiles. Here: $RPE_{B1} = 0.008$, and $EMD(h_{B2}, h_{B3}) = 0.008$; panel B shows example of trivial dynamic similarity.

References

[1] Hart, Y., Noy L., Feniger-Schaal R., Mayo A.E., Alon U.: (2014) Individuality and Togetherness in Joint Improvised Motion. PLoS ONE 9(2): e87213. doi:10.1371/ journal.pone.0087213

[2] Hogan, N., Flash, T.: (1987) Moving gracefully: quantitative theories of motor coordination. Trends in Neurosciences 10:170–174.

[3] Hogan, N., Sternad, D.: (2007) On rhythmic and discrete movements: reflections, definitions and implications for motor control. Exp Brain Res 181:13–30.

[4] Kilner, J.M., Paulignan, Y., Blakemore, S.J.: (2003). An Interference Effect of Observed Biological Movement on Action. Current Biology, 13(6), 522-525

[5] Kilner, J., Hamilton, A. F. d. C., Blakemore, S.J.: (2007). Interference effect of observed human movement on action is due to velocity profile of biological motion. Social Neuroscience, 2(3-4), 158-166.

[6] Noy, L., Dekel, E., Alon, U.: (2011) The Mirror Game as a Paradigm for Studying the Dynamics of Two People Improvising Motion Together. Proc Natl Acad Sci USA 108:20947–20952.

[7] Viviani, P., Flash, T.: (1995). Minimum-jerk, two-thirds power law, and isochrony: converging approaches to movement planning. Journal of Experimental Psychology: Human Perception and Performance, 21(1), 32.

[8] Levina, E., Bickel, P.: (2001)The earth mover's distance is the mallows distance: some insights from statistics. 2001 Eighth IEEE International Conference on Computer Vision, ICCV 2001 Proceedings vol. 2, 251–256.

[9] Kantorovich, L.V., Rubinstein, G.Sh.: (1958)On a space of completely additive functions (in Russian). Vestnik Leningrad Univ. 13(7), 52–59.

[10] Cohen, S., Guibas, L.: (1997) The earth mover's distance: Lower bounds and invariance under translation. DTIC Document, Tech. Rep.

[11] Billingsley, P.: Probability and Measure, Anniversary Edition. John Wiley and Sons, (2012)

[12] Johnson, N.L., Kotz, S., Balakrishnan, N.: (1994) Continuous Univariate Distributions, Volume 1, Wiley-Interscience.

[13] Borg, I., Groenen, P.J.F.: (2005) Modern Multidimensional Scaling: Theory and Applications. Springer Series in Statistics 2nd ed. 2005, 614 p.

[14] Slowinski, P., Rooke, E., di Bernardo, M., Tsaneva-Atanasova, K.: (2014) Kinematic characteristics of motion in the mirror game. IEEE International Conference on Systems, Man and Cybernetics, 748–753.

[15] Manly, B.F.: (2004) Multivariate statistical methods: a primer. CRC Press.

[16] Corder, G.W., Foreman, D.I.: (2014). Nonparametric Statistics: A Step-by-Step Approach. 2nd edition, Wiley.

[17] Equis, S., Jacquot, P.: (2010) Phase Extraction in Dynamic Speckle Interferometry with Empirical Mode Decomposition and Hilbert Transform, Strain 46, 550–558

[18] Varlet, M., Richardson, M.J.: (2011) Computation of continuous relative phase and modulation of frequency of human movement. Journal of biomechanics, 44(6), 1200–1204.

[19] Kralemann, B., Cimponeriu, L., Rosenblum, M., Pikovsky, A., Mrowka, R.: (2008) Phase dynamics of coupled oscillators reconstructed from data. Physical Review E, 77(6), 066205.

[20] Revzen, S., Guckenheimer, J.M.: (2008) Estimating the phase of synchronized oscillators. Physical Review E 78, 051907.

[21] (2015, March). [Online]. Available: http://www.stat.physik.uni-potsdam.de/ mros/damoco2.html

[22] Schmidt, R.C., Nie L., Franco A., Richardson, M.J.: (2014) Bodily synchronization underlying joke telling. Front. Hum. Neurosci. 8:633. doi: 10.3389/fnhum.2014.00633

[23] Issartel, J., Marin, L., Gaillot, P., Bardainne, T., Cadopi, M.: (2006). A practical guide on time-frequency analysis to study human motor behavior: the contribution of wavelet transform. J. Mot. Behav. 38, 139–159.

[24] Issartel, J., Bardainne, T., Gaillot P., Marin L.: (2015) The relevance of the cross-wavelet transform in the analysis of human interaction: a tutorial. Front. Psychol. 5:1566.

[25] Grinsted, A, Moore, J.C., Jevrejeva, S.: (2004) Application of the cross wavelet transform and wavelet coherence to geophysical time series. Nonlinear Processes in Geophysics 11, 561–566

[26] (2015, March). [Online]. Available: http://www.glaciology.net/wavelet-coherence

[27] Fisher, N.I.: (1995) Statistical Analysis of Circular Data. Cambridge Univeristy Press, Cambridge, UK

[28] Zhai, C., Alderisio, F., Tsaneva-Atanasova, K., di Bernardo, M.: (2014) Adaptive Tracking Control of a Virtual Player in the Mirror Game. Proceedings of IEEE Conference on Decision and Control (CDC), 7005–7010

[29] Zhai, C., Alderisio, F., Tsaneva-Atanasova, K., di Bernardo, M.: (2014) A novel cognitive architecture for a human-like virtual player in the mirror game. Proceedings of IEEE International Conference on Systems, Man and Cybernetics (SMC), 754–759.

[30] Pearson, K.: (1929) Editorial note to "Inequalities for moments of frequency functions and for various statistical constants". Biometrika 21 (1–4), 370–375. doi:10.1093/biomet/21.1-4.361.

[31] Flash, T., Hogan, N.: (1985) The coordination of arm movements: an experimentally confirmed mathematical model. J Neurosci 5:1688–1703.

[32] Fink, W.P., Kelso, S.J.A., Jirsa, V.K., de Guzman, G.: (2000) Recruitment of degrees of freedom stabilizes coordination. Journal of Experimental Psychology: Human Perception and Performance, 26(2), 671–692.

[33] Marin, L., Bardy, B.G., Bootsma, R.J.: (1999) Level of gymnastic skill as an intrinsic constraint on postural coordination, Journal of Sports Sciences, 17:8, 615-626.

[34] Schmidt, R.C., Carello, C., Turvey, M.T.: (1990) Phase transitions and critical fluctuations in the visual coordination of rhythmic movements between people. Journal of Experimental Psychology: Human Perception and Performance, 16(2), 227–47.
